# Effects of hyperglycemia on the regulation and expression of the *Apoe*, *Igfbp1* and *Foxo1* genes and on nuclear phenotypes in HepG2 cells

**DOI:** 10.1186/1756-8935-6-S1-P21

**Published:** 2013-03-18

**Authors:** Flávia G Ghiraldini, Maria Luiza S Mello

**Affiliations:** 1 Department of Structural and Functional Biology, Unicamp, Campinas, 13083-862, Brazil

## Background

The hyperglycemia caused by diabetes mellitus (DM) affects chromatin organization in hepatocytes of non-obese diabetic mice [1]. Sirt1, a NAD^+^-dependent deacetylase, has been related to cellular metabolic changes, acting as a nutritional sensor. Together with the transcription factors PGC-1α and Foxo1, Sirt1 affects chromatin conformation and the expression of genes involved in cell response to hyperglycemia [2].The aim of this study was to evaluate changes in the expression of the genes *Apoe*, *Igfbp1* and *Foxo1* and their possible epigenetic regulation by Sirt1-targets, and to assess chromatin remodeling in hepatoma HepG2 cells under hyperglycemic conditions.

## Materials and methods

HepG2 cells cultivated in an insulin-containing normoglycemic medium (NM) were used as a control (Group 1). Group 2 consisted of cells treated for 48 h with a high glucose medium (HGM) in absence of insulin, to simulate a type I DM condition. Cells of group 3 were treated similarly to those of group 2 and then returned to NM for 24 h. Cells treated with NM and HGM containing nicotinamide (NIC), a sirtuin inhibitor, for 6 h, represented groups 4 and 5, respectively. *Apoe*, *Igfbp1* and *Foxo1* expressions were evaluated by qPCR. The abundance of H3K9me2 and H3K9Ac markers in the promoters was analyzed by N-ChIP. Chromatin phenotypes were analyzed microspectrophotometrically in Feulgen-stained cells.

## Results

The gene expression in cells of the group 2 increased in comparison to those of group 1 but returned to basal levels in cells of group 3. Treatment with NIC in HGM (group 5) promoted gene expression similar only to that of group 2. When H3K9me2 and H3K9Ac marks on the promoters of these genes were analyzed, a progressive loss was detected in cells of groups 2 and 3, a general increase was found in cells of group 4, and no significant change occurred in cells of group 5. An increase in Feulgen-DNA amounts was found in cells from group 3. Treatment with NIC promoted increase in ploidy degrees and contrast between condensed and non-condensed chromatin.

## Conclusion

Changes in the expression of the studied genes, caused by hyperglycemia, showed to be rapidly modulated as a function of the cell culture medium composition. The sirtuin activity seemed to be inhibited by HGM, because of the similarity of results provided by treatment with NIC and HGM only. However, both H3K9Ac and H3K9me2 epigenetic marks presented a continuous response with HGM, indicating a different epigenetic signature. Moreover, the increase in DNA content in cells of the group 3 suggests induction of cell proliferation due to the stress conditions. Treatment with NIC showed that sirtuins play a role in chromatin remodeling in HepG2 cells.
